# The Management of “Coats' Response” in a Patient with X-Linked Retinitis Pigmentosa—A Case Report

**DOI:** 10.5402/2011/970361

**Published:** 2011-04-20

**Authors:** Shveta Bansal, Niladri Saha, W. H. Woon

**Affiliations:** ^1^Royal Preston Hospital, Sharoe Green Lane North, Fulwood, Preston, Lancashire PR29HT, UK; ^2^St James's University Hospital, Leeds LS97TF, UK

## Abstract

The management of exudative retinal detachment in Coats disease can be very difficult with variable results. A case is presented of a 12 year old boy who was diagnosed with X-linked retinitis pigmentosa with an associated “Coat's Response”. The patient had a marked reduction in his left visual acuity due to intragel and subhyaloid haemorrhage as well as exudative retinal detachment. This was managed successfully with vitrectomy and endolaser, resulting in clearance of the haemorrhage and flattening of the retina. In our experience endolaser should be considered as viable therapeutic option in the management of this condition.

A 12-year-old boy with a family history of X-linked retinitis pigmentosa (XLRP) presented with nyctalopia, restricted peripheral vision and difficulties at school. There were no other significant medical abnormalities; however, there was a known family history of X-linked retinitis pigmentosa. On examination, best corrected visual acuity was 6/18+1 in each eye. There was bone spicule pigmentation in the peripheral retina of both eyes. In the left eye, cystoid macular oedema was detected with yellowish exudation and telangectasia in the inferior retina (Figures 1 and 2). Goldmann's visual fields showed constriction. The patient was initially managed with oral Diamox (250 mg SR bd), however; cystoid macular oedema did not improve.

Electrodiagnostic tests showed findings consistent with retinitis pigmentosa, and a diagnosis of XLRP based on the family pedigree and genetic analysis was made along with an exudative retinopathy (Coats' response) in the left eye. The following year, the patient presented with reduced visual acuity in the left eye (R 6/18; L 6/60). There was a subhyaloid and intragel haemorrhage with exudative retinal detachment (Figure 3). The haemorrhage failed to resolve over the ensuing months, and a decision was made, after informed consent, to proceed to vitrectomy. During surgery, it was not possible to obtain a cryotherapy reaction in the area of telangiectatic vessels, despite several attempts. In view of this, endolaser was performed. One week and 3 months postoperatively, although there was no improvement in the visual acuity, a marked reduction in the exudates was noticed and the retina remained flat (Figure 4).

Since the original description of Coats' disease in 1908, a number of other conditions have been identified in which a-Coats' type response is observed in both adults and children. This response has been previously reported to occur in retinitis pigmentosa with an incidence of up to 3.6% in those affected; however, the management of such patients remains difficult and disputed [1]. 

In early Coats' disease, photocoagulation or cryotherapy of telangiectatic vessels has been advocated by various groups suggesting that aggressive management prevents disease progression and visual loss [2]. In advanced cases (stages 3 and 4), the focus of management shifts from preserving vision to preserving a painless eye. In these cases, the conventional wisdom has been to perform vitrectomy, scleral buckling cryotherapy, and drainage of the exudate via either a retinotomy or external drain [3]. 

Laser in advanced Coats' disease has been deemed less successful. In our experience, repeated attempts at cryopexy were unsuccessful during the procedure. Endolaser, however, produced good results. Yoshizumi et al. observed similar findings in one advanced exudative case managed with external drainage and attempted cryotherapy then subsequent laser photocoagulation [3]. They suggest that retained cholesterol deposits insulated the retinal telangectasia preventing freezing. Furthermore, Nucci et al. describe a series of 32 patients with advanced disease managed with laser alone resulting in better or stable vision in all but one case [4].

In our experience, endolaser was more effective than cryotherapy perhaps for the reasons stated above. We propose that it should be considered a viable therapeutic option in the management of this difficult condition.

## Figures and Tables

**Figure 1:**
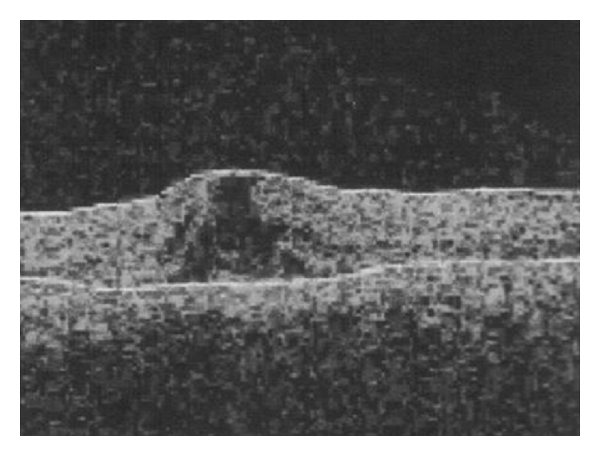
Optical coherence tomography scan of the left eye showing macular oedema.

**Figure 2:**
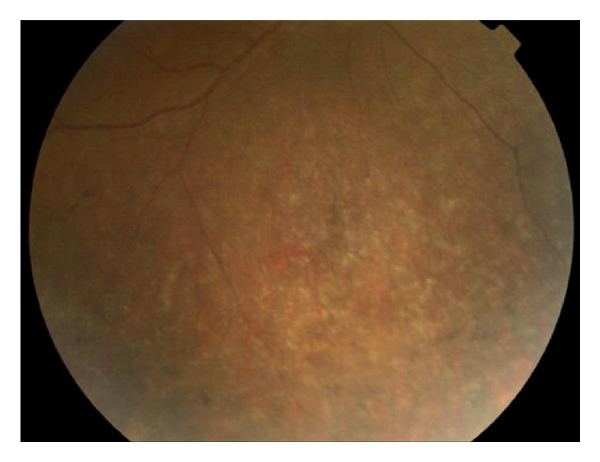
Retinal photograph of the inferior retina of the left eye showing a bone spicule pattern of pigmentation and peripheral exudates.

**Figure 3:**
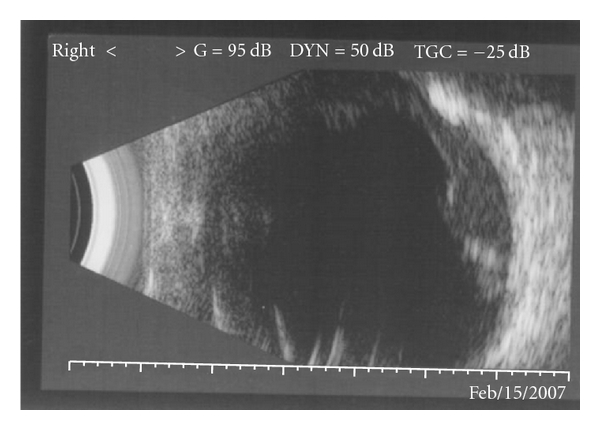
Ultrasound B scan of the left eye showing a subhyaloid haemorrhage and overlying retinal detachment.

**Figure 4:**
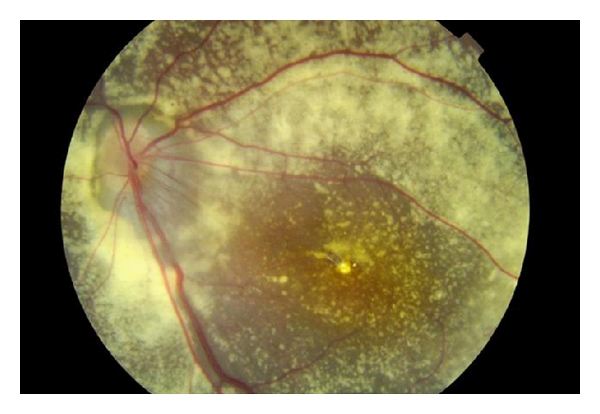
Retinal photograph of the left eye three months after surgery. The retina remains flat.
